# Adaptation Mechanisms of Yak (*Bos grunniens*) to High-Altitude Environmental Stress

**DOI:** 10.3390/ani11082344

**Published:** 2021-08-09

**Authors:** Wondossen Ayalew, Min Chu, Chunnian Liang, Xiaoyun Wu, Ping Yan

**Affiliations:** 1 Key Laboratory of Yak Breeding Engineering, Lanzhou Institute of Husbandry and Pharmaceutical Sciences, Chinese Academy of Agricultural Sciences, Lanzhou 730050, China; chumin@caas.cn (M.C.); chunnian2006@163.com (C.L.); 2 Department of Animal Production and Technology, Wolkite University, Wolkite P.O. Box 07, Ethiopia

**Keywords:** adaptation, high altitude, hypoxia, yak

## Abstract

**Simple Summary:**

The yak is a multipurpose domesticated animal that serves as a protein source for local herders and a sacred carrier of culture and religion. Besides their economic significance, yaks harbor special morphological, physiological, biochemical, and genetic adaptations for tolerance to high-altitude stress. Morphologically, yaks have large hearts and lungs, compact bodies, thick outer hair covering, and nonfunctional sweat glands, which help to withstand hypoxia and cold stress. A reduced heat production, decreased respiration and sweating, reduced metabolism, and efficient nitrogen utilization are the major physiological and biochemical mechanisms for yak survival at high altitudes. Furthermore, the yak has undergone long-term natural selection and developed a unique genetic architecture that favors survival in hostile environments. The yak expresses the HIF-1α pathway-related genes (*ADAM17*, *ARG2*, and *MMP3*) putatively involved in hypoxia response and nutrition pathways genes (*CAMK2B*, *GENT3*, *HSD17B12*, *WHSC1*, and *GLUL*) for nutritional assimilation at high altitudes.

**Abstract:**

Living at a high altitude involves many environmental challenges. The combined effects of hypoxia and cold stress impose severe physiological challenges on endothermic animals. The yak is integral to the livelihood of the people occupying the vast, inhospitable Qinghai–Tibetan plateau and the surrounding mountainous region. Due to long-term selection, the yak exhibits stable and unique genetic characteristics which enable physiological, biochemical, and morphological adaptations to a high altitude. Thus, the yak is a representative model for mammalian plateau-adaptability studies. Understanding coping mechanisms provides unique insights into adaptive evolution, thus informing the breeding of domestic yaks. This review provides an overview of genetic adaptations in *Bos grunniens* to high-altitude environmental stress. Combined genomics and theoretical advances have informed the genetic basis of high-altitude adaptations.

## 1. Introduction

The Qinghai–Tibetan Plateau ecological environment is characterized by low atmospheric oxygen pressure, cold, and limited feed supplies [1,2]. Endothermic animals endure impaired oxygen supplies, which compromise cellular functions and physiologic performance under high-altitude environments. These high-altitude mountains are more sensitive and vulnerable to climate change, a huge threat to biodiversity and the ecosystem [3]. Moreover, the cold temperature increases the environmental harshness with the temperature dropping by ~6 °C per kilometer above sea level [4]. Other factors, including late winter and early spring, feed shortage, and snow cover, inevitably lead to severe malnutrition and weight loss among animals [5,6]. Species have developed special characteristic features through natural selection to adapt to extreme terrestrial environments [7,8].

The yaks are the world’s most remarkable domestic animal living freely and reproducing under the harsh plateau environment [1]. The yak was domesticated >7300 years ago from wild yak by the early nomadic people and is the only large animal that coexists with its wild ancestors in a similar environment [9]. Over 17.6 million yaks exist globally, and the majorities are found in the plateau regions of central Asia, which cover ~2.5 million km^2^ centered around the Qinghai–Tibetan Plateau and adjacent highlands [10]. The yak supplies (milk, meat, hair, hides, and manure) and services (draft, packing, and riding) the pastoralists and agro-pastoralists occupying these areas. They are also means of financial security and cultural functions (status, dowry, religious festivals) [6]. They also offer a good framework for studying the effects of natural and artificial selection in livestock domestication and adaptation to different environments.

The yak is the only bovine species native to the Qinghai–Tibetan Plateau and adjacent highlands that exhibits a high adaptability to high altitudes, a low sensitivity to cold, a low oxygen pressure, and prolonged periods (approximately half a year) of food scarcity [3,6,11,12]. Natural and artificial selections from domesticated yaks resulted in breeds with distinct morphological, physiological, and adaptability traits that enhanced survival in harsh environments [13,14,15]. Understanding the aerobic metabolism of yaks under hypoxic environments can provide important insights into adaptive evolution [16]. Together with advanced molecular techniques and genetic research, these insights provide a basis for investigating the genetic mechanisms underlying adaptability to climate change, the current research hotspot [17,18]. 

Knowledge of the mechanisms underlying adaptation to various agro-ecosystems is essential for the effective management of farm animal genetic resources [7,19]. Intriguingly, multiple studies have focused on yak adaptation, allowing researchers to understand the morphological, physiological, biochemical, and genetic mechanisms of adaptation to extremely high altitudes [13,15,20,21,22]. Although research on the adaptation mechanisms of the yak in high altitudes has increased exponentially, review studies on the comprehensive, adaptive mechanisms remain scarce. Therefore, this review attempts to collate and synthesize current knowledge on the mechanisms of yak adaptation to high altitudes. Furthermore, it can also provide new avenues for in vitro and in vivo studies to further test hypotheses arising from previous investigations and options for designing and implementing interventions for improved yak productivity and resilience in high altitudes.

## 2. High Altitude Adaptation Mechanisms of Yak

**Figure 1:**
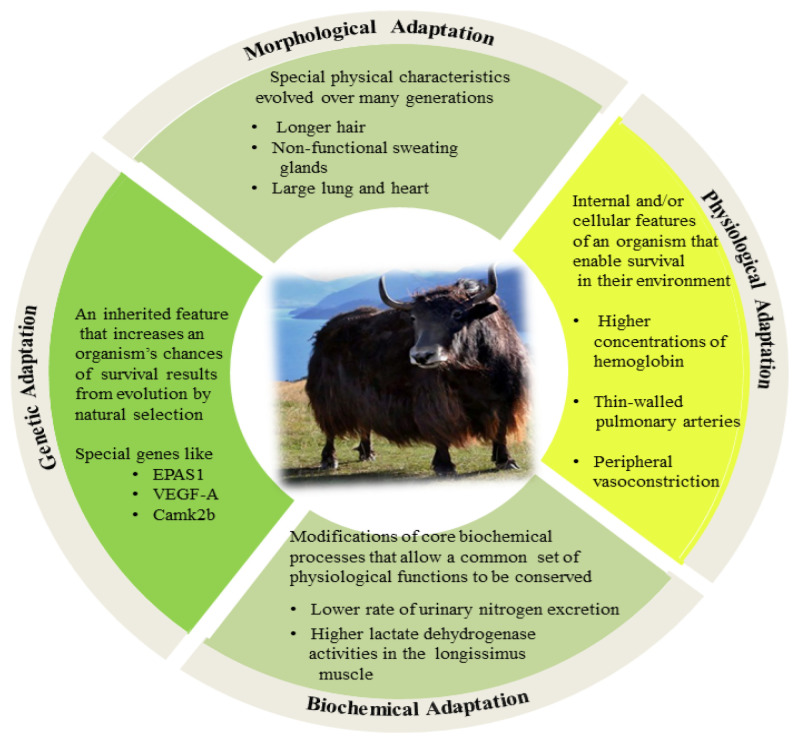
Schematic representation of the yak adaptation to high-altitude environmental stress in comparison to a closely related species (*Bos indicus* or cattle).

High altitudes negatively impact the normal bodily functions of individuals, whether they are accustomed or unaccustomed to such environments. Mishra and Ganju reviewed high-altitude environmental factors, such as cold and hypobaric hypoxia, which affect the immune system, making it more susceptible to cancer, infection, and autoimmune disease [23]. Inadequate hypoxia treatment affects reproduction and fertility traits, including reduced intrauterine growth in sheep [24] and impaired development and function of corpus luteum [25]. There should be an increased focus on breeding and managing animals for an improved resilience to applied stressors [26]. To adapt to high-altitude environments, plateau-dwelling mammals have developed some distinct characteristics. The yak, a unique breed that inhabits the alpine pastoral area of the Tibetan Plateau, is one of the rare bovine breeds adapted to high altitudes and cold climates [12,27]. The adaptive process is extremely complex, consisting of several components that exhibit stable and unique genetic characteristics for regulating the physiological, biochemical, and morphological mechanisms of adaption to a high altitude (Figure 1).

### 2.1. Morphological Adaptations

Morphological adaptations are physical changes that occur over many generations of animals to enhance fitness in a given environment. Over many generations, the native high-altitude *B. grunniens* successfully adapted to the chronic hypoxia of high altitudes despite belonging to the genus *Bos*, closely related to cattle [28]. The exceptional adaptation of the yak to high altitudes is related to evolved special morphological mechanisms (Table 1). Compared with close relatives such as cattle that live at lower altitudes, yaks have relatively larger lungs and hearts [29]. Furthermore, the yak has longer, wider, and rounder pulmonary-artery endothelial cells with little smooth muscles, which allows for improved functioning in high-altitude environments compared to cattle [28,30].

Not only does the hypoxic environment impact life at high altitudes, freezing temperatures and scarce food supply also contribute to the harsh environment. The alpine habitat of yaks is at altitudes of 3000–6000 m. As a result, there is no frost-free period throughout the year. The yaks are well-adapted to the cold, high-altitude environment; their bodies are compact with a relatively reduced skin surface area per unit of body weight (0.016 m^2^/kg). They also have thick outer hair coats and no functional sweat glands [6]. The absence of sweating in the yak enhances cold tolerance [32].

Furthermore, the thick fleece covering the entire yak body enhances heat conservation. The thick fleece comprises an outer coat of long hair and an undercoat consisting of a dense layer of fine down fibers that appear in the colder season to contain body heat and repel moisture [6]. Undoubtedly, feeding mechanisms are an important factor in determining the success and survival of vertebrate species within their environment [33]. The alpine habitats at high altitudes are marked by a severe climate and very short growing seasons with limited grazing resources and often treacherous terrain. Together, these factors lead to severe malnutrition and weight loss among animals [6,34]. Yaks have developed shorter tongues with greater lingual prominence, larger and more numerous conical papillae, and thicker keratinized epithelium than domestic cattle. These attributes enable the yak to consume a wider variety of pasture plant species [31]. Furthermore, the yak rumen is unusually large relative to omasum. This large rumen allows it to consume large quantities of low-quality food at a time and to ferment it longer in order to extract more nutrients during times of nutritional scarcity [6].

### 2.2. Physiological Adaptations

Physiology can be viewed as mechanisms and processes that allow organisms to deal with internal challenges (for example, exercise, growth, and reproduction) and external stress (e.g., variations in temperature, oxygen, water availability, salinity, pressure, radiation, and heavy metals, etc.). The yak inhabits the entire Qinghai–Tibetan Plateau, and physiological adaptations have contributed to their success in surviving hostile environmental conditions [35]. Chronic hypoxia is the primary stressor of high-altitude conditions and limits the efficient functioning of respiratory and cardiovascular systems in mammals and birds [36]. Interestingly, the prolonged exposure of yaks to high altitudes increases their physiological response to chronic hypoxia because yaks have a larger pulmonary alveolar area per unit area, thinner alveolar septum, thinner blood–air barrier [37], larger hearts and lungs, and higher concentrations of erythrocytes and hemoglobin than other cattle species [29]. Thin-walled pulmonary arteries with little smooth muscle and absent right ventricular hypertrophy are additional hypoxic adaptations observed in yaks [16]. Indeed, these characteristics and changes in the cardiovascular system compensate for the hypobaric high-altitude environment [5].

Furthermore, this adaptation is probably due to natural selection, which enhances the hypoxic pulmonary vasoconstrictor to respond with no hypoxemic stimulus for increased red blood cell production and hemoglobin concentrations [16]. Previous research demonstrates that chronic exposure to hypoxic conditions raises the blood/erythrocyte volume for high-altitude native animals [38]. As a result, hyperventilation, hemoconcentration, and stimulated erythropoiesis are physiological responses that warrant oxygen delivery to tissues [39,40].

Adaptation often occurs at the expense of performance, and survivability is often better in “low” performance animals because of their low input needs (especially feed) and moderate internal heat production [41]. In their natural habitat, yaks must maintain normal energy production under hypoxic conditions [5,11] and optimize nutritional assimilation as a consequence of the cold stress [5] and limited feed [6]. Under cold stress, heat loss is prevented by peripheral vasoconstriction and heat production through shivering and uncoupled mitochondrial activity [42]. Furthermore, multiple cold-adapted species can temporarily slow their metabolisms in response to harsh environmental conditions, leading to torpor or, in extreme cases, hibernation [43]. The significant reduction in heat production of yaks during winter might result from their adaptation to low oxygen concentrations in the air, the cold environment, and the long-term under nutrition prevalent in the six-months-long cold season of the Tibetan plateau [5].

### 2.3. Biochemical Adaptations 

The cold, hypoxic conditions of high-altitude habitats impose severe metabolic demands on endothermic vertebrates. Understanding how high-altitude endotherms cope with the combined effects of hypoxia and cold can provide important insights into the process of adaptive evolution. Biochemical adaptions provide fascinating insights into how organisms “work” and how they evolve to sustain physiological function under a vast array of environmental conditions. Their high blood hemoglobin concentrations enable yaks to adapt to tolerating low atmospheric partial pressures of oxygen characteristic of the >2000 m Qinghai–Tibetan plateau [5,6]. The lower energy metabolism of yaks might result from adaptation to a low O_2_ concentration in the air, a cold environment, and the long-term undernutrition during the annual six-months-long cold season of the Qinghai–Tibetan plateau. The yak rumen microorganisms ferment approximately 70–80% of the feed intake and produce volatile fatty acids, providing 60–75% of the required metabolic energy. This phenomenon may be a coevolutionary coping strategy for low feed resources in cold environments [44]. Compared to indigenous cattle, yaks have a lower rate of urinary nitrogen excretion (a possible adaptation to a poor feed supply) and more efficient nitrogen utilization, which is at least in part due to a greater microbial protein production in the rumen [45]. This adaptability is believed to assist in the rapid recovery of body weight over the summer grazing period [46,47]. In addition, the low maintenance protein requirements [48] and low surface area of the yak body [49] result in a low metabolic rate. Altogether, these attributes are beneficial for the yak’s survival in the harsh environmental conditions of the Tibet plateau. 

Despite variations among species, most studies attribute metabolic adaptation under high altitudes to a decreased muscle oxidative capacity. In this regard, lactate dehydrogenase (LDH) is the crucial enzyme in anaerobic glycolysis, catalyzing the conversion between pyruvate and lactate, a critical role in energy metabolism [50]. Interestingly, unlike cattle, the yak exhibits higher LDH activities in the longissimus muscle, facilitating carbohydrate utilization under limited oxygen supplies. Thus, this provides a unique adaptive feature of yaks in high altitudes [51].

### 2.4. Genetic Background of High-Altitude Adaptations

Genetic adaptations to novel environments and climatic changes are a fundamental process for species’ survival. The genetic mechanism for adaption to high altitudes appears to be more complicated than any other phenotype understood thus far [52]. Species have developed special characteristic features through long-term selection to cope with the specific stressors of extreme terrestrial environments [7,8]. Genetic variation in a population provides flexibility to adapt to changing environments and is crucial for the survival and speciation of that population over time [53]. In the past, it was customary to focus on structural sequence variation and consider each gene as a separate unit of evolution in population genetic theory and empirical practice. However, adaptive phenotypes are more likely a function of polygenic mutations. The detection of adaptive genetic signals with conventional selection methods is more complicated than detecting other phenotypes [54]. It is widely accepted that adaptive evolutionary mechanisms can evolve via changes at one or a few loci (selective sweep) with major effects or via simultaneous allele frequency shifts at many loci with small effects [55]. The major developments in sequencing and genotyping technology over the past decade have facilitated the identification and selection of population-specific genome signatures for livestock adaptation. In the yak populations, several genes putatively associated with adaptation to life at high altitudes were identified. These genes are primarily related to physiological pathways in response to hypoxia and temperature acclimatization and modifications of the cardiovascular system and energy metabolism [13,20,56]. Endothelial PAS domain-containing protein 1 (*EPAS1)* is the top candidate gene encoding the hypoxia-inducible transcription factor (*HIF-2α*). This gene (*EPAS1*) is believed to regulate erythropoietin production, which changes with the available oxygen in the cellular environment under high-altitude conditions [13,21,57]. There are also distinct selection signatures within yaks, which suggest unique adaptation mechanisms (Table 2).

For example, the vascular endothelial growth factor-A (*VEGF-A*) gene is a key regulator of angiogenesis and an endothelial cell mitogen that regulates blood vessel size as an adaptation to high-altitude functioning [35]. Furthermore, yaks must not only maintain a normal energy production under hypoxic conditions [5,11] but must also optimize nutritional assimilation as a consequence of the limited forage resources available in their high-altitude environments [6]. Qiu et al. [13] reported five key genes showing positive selection in the yak nutritional and metabolic pathways. Among the five described genes, *Camk2b* gene regulates the secretion of gastric acid in the rumen, contributing to the assimilation of volatile fatty acids produced by ruminal fermentation [58,59]. In addition, *Gcnt3*, *Hsd17b12*, *Whsc1,* and *Glul* are functional in the polysaccharide, fatty acid, and amino-acid metabolism, respectively [60,61]. The positively selected changes in *Glul* may be important for the enhanced nitrogen utilization in yaks [11]. Other genes putatively involved in the yak adaptation to high-altitude environments are summarized in Table 2.

## 3. Transcriptomic Changes in Yaks Living in High-altitude Environments

Gene expression profiles indicate the activation of specific molecular pathways/networks of genes that regulate external stimuli and provide insight into the role of regulatory variation in adaptive evolution [67,68,69]. Recent studies have identified different genes and pathways that widely participate in various biological processes, including adaptations to hypoxia. During periods of reduced O_2_ availability, changes in gene expression are mediated by a specific family of transcription factors called the “master regulator” of O_2_ homeostasis. These genes are collectively known as hypoxia-inducible factors (*HIF*) [70,71]. Hypoxia-inducible factors are oxygen-dependent transcriptional activators, crucial in tumor angiogenesis and mammalian development through the transcriptional regulation of oxygen homeostasis genes in response to hypoxia [72]. In mammalian cells, *HIFα* isoforms (*HIF-1α* and *HIF-2α*) are the most extensively studied and understood central mediators of cellular adaptation to hypoxia. *HIF1α* functions as a key regulator of O_2_ homeostasis that coordinates oxygen sensing and intracellular responses to hypoxia by regulating the expression of hundreds of genes. Some of the *HIF1α*-regulated genes belong to biological pathways for energy metabolism, angiogenesis, erythropoiesis, iron homeostasis, and apoptosis [73,74,75].

Because of their adaptation to high-altitude environments coupled with exceptional physical endurance, yaks ought to be a model animal for understanding the molecular basis of high-altitude adaptation. As a result, the cDNA of *HIF-1α*, a subunit of *HIF-1β* in the yak, was sequenced by Dolt et al. [57]. The authors observed the variant-specific expression of *HIF-1α* in the blood and liver. In contrast, expression was absent in the lung, heart, and kidney of yaks. This tissue-specific expression might be the consequence of the alternative splicing of *HIF-1α*, as observed in plateau pika, another high-altitude-adapted animal [76].

Furthermore, a comparison of tissue-specific expression between yaks and cattle revealed that *HIF-1α* was ubiquitously expressed, whereas *HIF-2α* expression in the yak was limited to endothelial cells in tissues (kidney, heart, lung, spleen, and liver) and blood [72]. The expression of both *HIF-1α* and *HIF-2α* was higher in the yak tissues than in cattle. Other comparative transcriptome studies between cattle and yaks revealed that, among several organs, the gene expression patterns of the heart showed the greatest differentiation between the two species [77] and that differentially expressed genes in lung and gluteus tissue were involved in red blood cell development and inhibition of blood coagulation [78].

## 4. Conclusions

High-altitude habitats are characterized by extremely harsh climates consisting of low temperature and low oxygen pressure. The native high-altitude yak has evolved multiple unique adaptations, including morphological, physiological, biochemical, and genetic changes due to long-term selection. To gain a more holistic understanding of high-altitude adaptations, these types of studies need expansion, and efforts should be made to integrate work on DNA sequence polymorphism with analyses of transcriptional variation. Indeed, cold and hypoxia act synergistically on an organism’s performance at high altitudes, yet the vast majority of studies have focused solely on adaptation to hypoxia. Hence, joint investigations of these co-occurring environmental stressors should be the priority for future research. Furthermore, the current climate change scenario characterized by rising temperature undoubtedly alters the natural habitats of yaks by creating new environmental conditions to which these animals were never before exposed. Therefore, further investigations to determine how these shifts in climate contribute to changes in the yak production and the livelihoods of highlanders are indispensable.

## Figures and Tables

**Table 1:** Key morphological adaptations of yaks to high-altitude environments.

Special Morphological Structures	Function	References
Compact body, thick outer hair covering, and nonexistence of functional sweat glands	Minimize dissipation of body heat during winter	[6]
Thin-walled pulmonary arteries with little smooth muscles	Facilitate superefficient O_2_ flow under hypobaric hypoxia	[16]
Larger lungs and hearts	Aid oxygen uptake	[17]
Shorter tongue and greater lingual prominence	Improve forage digestibility through efficient grinding of food	[31]

**Table 2:** Genes/candidate genes underlying selection signatures of yak adaptation to high altitudes.

Candidate Genes	Functions	References
*Camk2b*, *Gcnt3*, *Hsd17b12*, *Whsc1,* and *Glul*	High level of nutrition utilization in high altitudes	[13]
*HIF1A*, *MMP3*, *ADAM17*, *ARG2*	High-altitude adaptation	[13]
*DEXI*, *DCC,* and *MRP4*	Adaptation to high-altitude environments	[14]
*PDE4D*, *RPS6KA6*, *ITPR1,* and *GNAO1*	Environmental information processing and environmental adaptability	[20]
*EPAS1*	Key transcription factor that activates the expression of oxygen-regulated genes	[21]
*ABCG8*, *COL4A1*, *LOC102287650*, *PDCD1*, and *NUP210*	Adaptation to high-altitude environments	[22]
*VEGF-A*	Regulation of blood vessel size	[35]
*MMP3*	Regulator of the cellular response to hypoxia	[58]
*HIF-1α*	Transcription of genes involved in oxygen homeostasis	[59]
*AQP4*	Resistance to cerebral edema	[62]
*ATP8* and *ATP6*	Mitochondrial ATPase assembly	[63]
*DCC*, *GSTCD*, *MRPS28,* and *MOGAT2*	Adaptation to high-altitude environments	[64]
*MT-ND1* and *MT-ND2*	Electron transport chain of oxidative phosphorylation	[65]
*GRIK4*, *IFNLR1*, *LOC102275985*, *GRHL3*, and *LOC102275713*	Physiological regulation under a hypoxic environment	[66]

## References

[1] Miao F., Guo Z., Xue R., Wang X., Shen Y. (2015). Effects of Grazing and Precipitation on Herbage Biomass, Herbage Nutritive Value, and Yak Performance in an Alpine Meadow on the Qinghai–Tibetan Plateau. PLoS ONE.

[2] West J.B. (2015). Recent Advances in High Altitude Medicine and Biology. High Alt. Med. Biol..

[3] Ghatak D., Sinsky E., Miller J. (2014). Role of snow-albedo feedback in higher elevation warming over the Himalayas, Tibetan Plateau and Central Asia. Environ. Res. Lett..

[4] Vuille M., Singh V.P., Singh P., Haritashya U.K. (2011). Climate variability and high altitude temperature and precipitation. Encyclopedia of Snow, Ice and Glaciers.

[5] Han X.T., Xie A.Y., Bi X.C., Liu S.J., Hu L.H. (2002). Effects of high altitude and season on fasting heat production in the yak *Bos grunniens* or *Poephagus grunniens*. Br. J. Nutr..

[6] Wiener G., Han J., Long R. (2003). The Yak.

[7] Key N., Sneeringer S. (2014). Potential Effects of Climate Change on the Productivity of U.S. Dairies. Am. J. Agric. Econ..

[8] Burtscher M., Gatterer H., Burtscher J., Mairbäurl H. (2018). Extreme Terrestrial Environments: Life in Thermal Stress and Hypoxia. A Narrative Review. Front. Physiol..

[9] Qiu Q., Wang L., Wang K., Yang Y., Ma T., Wang Z., Zhang X., Ni Z., Hou F., Long R. (2015). Yak whole-genome resequencing reveals domestication signatures and prehistoric population expansions. Nat. Commun..

[10] Ma Z.-J., Zhong J.-C., Han J.-L., Xu J.-T., Liu Z.-N., Bai W.-L. (2013). Research progress on molecular genetic diversity of the yak (*Bos grunniens*). Yi Chuan Hered..

[11] Wang H., Long R., Liang J.B., Guo X., Ding L., Shang Z. (2011). Comparison of Nitrogen Metabolism in Yak (*Bos grunniens*) and Indigenous Cattle (*Bos taurus*) on the Qinghai-Tibetan Plateau. Asian-Australas. J. Anim. Sci..

[12] Lan D., Xiong X., Huang C., Mipam T.D., Li J. (2016). Toward understanding the genetic basis of yak ovary reproduction: A characterization and comparative analyses of estrus ovary transcriptiome in yak and cattle. PLoS ONE.

[13] Qiu Q., Zhang G., Ma T., Qian W., Wang J., Ye Z., Cao C., Hu Q., Kim J., Larkin D.M. (2012). The yak genome and adaptation to life at high altitude. Nat. Genet..

[14] Zhang Z., Xu D., Wang L.I., Hao J., Wang J., Zhou X., Wang W., Qiu Q., Huang X., Zhou J. (2016). Convergent evolution of rumen microbiomes in high-altitude mammals. Curr. Biol..

[15] Lan D., Xiong X., Ji W., Li J., Mipam T.D., Ai Y., Chai Z. (2018). Transcriptome profile and unique genetic evolution of positively selected genes in yak lungs. Genetica.

[16] Ding X., Liang C., Guo X., Wu X., Wang H., Johnson K., Yan P. (2014). Physiological insight into the high-altitude adaptations in domesticated yaks (*Bos grunniens*) along the Qinghai-Tibetan Plateau altitudinal gradient. Livest. Sci..

[17] Yang J., Li W.-R., Lv F.-H., He S.-G., Tian S., Peng W.-F., Sun Y.-W., Zhao Y.-X., Tu X.-L., Zhang M. (2016). Whole-Genome Sequencing of Native Sheep Provides Insights into Rapid Adaptations to Extreme Environments. Mol. Biol. Evol..

[18] Friedrich J., Wiener P. (2020). Selection signatures for high-altitude adaptation in ruminants. Anim. Genet..

[19] Rojas-Downing M.M., Nejadhashemi A.P., Harrigan T., Woznicki S.A. (2017). Climate change and livestock: Impacts, adaptation, and mitigation. Clim. Risk Manag..

[20] Guang-Xin E., Basang W.D., Zhu Y.B. (2019). Whole-genome analysis identifying candidate genes of altitude adaptive ecological thresholds in yak populations. J. Anim. Breed. Genet..

[21] WU X.Y., DING X.Z., Min C.H.U., Xian G.U.O., BAO P.J., Liang C.N., Ping Y.A.N. (2015). Novel SNP of EPAS1 gene associated with higher hemoglobin concentration revealed the hypoxia adaptation of yak (*Bos grunniens*). J. Integr. Agric..

[22] Guang-Xin E., Yang B.-G., Basang W.-D., Zhu Y.-B., An T.-W., Luo X.-L. (2019). Screening for signatures of selection of Tianzhu white yak using genome-wide re-sequencing. Anim. Genet..

[23] Mishra K.P., Ganju L. (2010). Influence of High Altitude Exposure on the Immune System: A Review. Immunol. Investig..

[24] Parraguez V.H., Atlagich M., Díaz R., Bruzzone M.E., Behn C., Raggi L.A. (2005). Effect of hypobaric hypoxia on lamb intrauterine growth: Comparison between high- and low-altitude native ewes. Reprod. Fertil. Dev..

[25] Parraguez V.H., Urquieta B., Perez L., Castellaro G., De los Reyes M., Torres-Rovira L., Aguado-Martínez A., Astiz S., González-Bulnes A. (2013). Fertility in a high-altitude environment is compromised by luteal dysfunction: The relative roles of hypoxia and oxidative stress. Reprod. Biol. Endocrinol..

[26] Colditz I.G., Hine B.C. (2016). Resilience in farm animals: Biology, management, breeding and implications for animal welfare. Anim. Prod. Sci..

[27] Fu M., Chen Y., Xiong X., Lan D., Li J. (2014). Establishment of Mammary Gland Model In Vitro: Culture and Evaluation of a Yak Mammary Epithelial Cell Line. PLoS ONE.

[28] Durmowicz A.G., Hofmeister S., Kadyraliev T.K., Aldashev A.A., Stenmark K.R. (1993). Functional and structural adaptation of the yak pulmonary circulation to residence at high altitude. J. Appl. Physiol..

[29] Guan J., Long K., Ma J., Zhang J., He D., Jin L., Tang Q., Jiang A., Wang X., Hu Y. (2017). Comparative analysis of the microRNA transcriptome between yak and cattle provides insight into high-altitude adaptation. PeerJ.

[30] Wang D.P., Li H.G., Li Y.J., Guo S.C., Yang J., Qi D.L., Jin C., Zhao X.Q. (2006). Hypoxia-inducible factor 1α cDNA cloning and its mRNA and protein tissue specific expression in domestic yak (*Bos grunniens*) from Qinghai-Tibetan plateau. Biochem. Biophys. Res. Commun..

[31] Shao B., Long R., Ding Y., Wang J., Ding L., Wang H. (2010). Morphological adaptations of yak (*Bos grunniens*) tongue to the foraging environment of the Qinghai-Tibetan Plateau1. J. Anim. Sci..

[32] Krishnan G., Paul V., Hanah S.S., Bam J., Das P.J. (2016). Effects of climate change on yak production at high altitude. Indian J. Anim. Sci..

[33] Roth G., Wake D.B., Wake D.B., Roth G. (1989). Conservatism and innovation in the evolution of feeding in vertebrates. Complex Organismal Functions: Integration and Evolution in Vertebrates.

[34] Long R.J., Ding L.M., Shang Z.H., Guo X.H. (2008). The yak grazing system on the Qinghai-Tibetan plateau and its status. Rangel. J..

[35] Wu X.Y., Liang C.N., Ding X.Z., Guo X., Bao P.J., Chu M., Liu W.B., Yan P. (2013). Association of novel single-nucleotide polymorphisms of the vascular endothelial growth factor-A gene with high-altitude adaptation in yak (*Bos grunniens*). Genet. Mol. Res..

[36] Ivy C.M., Scott G.R. (2015). Control of breathing and the circulation in high-altitude mammals and birds. Comp. Biochem. Physiol. Part A Mol. Integr. Physiol..

[37] Wei Q., Yu H. (2008). Comparison of histological structure of pulmonary alveoli between 180 days old yak and plain cattle. J. Qinghai Univ. Nat. Sci..

[38] Claydon V.E., Norcliffe L.J., More J.P., Rivera M., Leon-Velarde F., Appenzeller O., Hainsworth R. (2004). Orthostatic tolerance and blood volume in Adens high-altitude dwellers. Exp. Physiol..

[39] West J.B. (2006). Human responses to extreme altitudes. Integr. Comp. Biol..

[40] West J.B. (2017). Physiological Effects of Chronic Hypoxia. N. Engl. J. Med..

[41] Gaughan J.B., Sejian V., Mader T.L., Dunshea F.R. (2019). Adaptation strategies: Ruminants. Anim. Front..

[42] Manou-Stathopoulou V., Goodwin C.D., Patterson T., Redwood S.R., Marber M.S., Williams R.P. (2015). The effects of cold and exercise on the cardiovascular system. Heart.

[43] Ruf T., Geiser F. (2015). Daily torpor and hibernation in birds and mammals. Biol. Rev..

[44] Zou H., Hu R., Wang Z., Shah A.M., Zeng S., Peng Q., Xue B., Wang L., Zhang X., Wang X. (2019). Effects of Nutritional Deprivation and Re-Alimentation on the Feed Efficiency, Blood Biochemistry, and Rumen Microflora in Yaks (*Bos grunniens*). Animals.

[45] Zhou J.W., Zhong C.L., Liu H., Degen A.A., Titgemeyer E.C., Ding L.M., Shang Z.H., Guo X.S., Qiu Q., Li Z.P. (2017). Comparison of nitrogen utilization and urea kinetics between yaks (*Bos grunniens*) and indigenous cattle (*Bos taurus*). J. Anim. Sci..

[46] Han X.T., Chen J., Han Z.K. (1998). Ruminal nitrogen metabolism and the flows of nitrogen fractions reaching the duodenum of growing yaks fed diets containing different levels of crude protein. Acta Zoonutrimenta Sin..

[47] Wang H.C., Long R.J., Zhou W., Li X.P., Zhou J.W., Guo X.S. (2009). A comparative study on urinary purine derivative excretion of yak (*Bos grunniens*), cattle (*Bos taurus*), and crossbred (*Bos taurus* x *Bos grunniens*) in the Qinghai-Tibetan plateau, China. J. Anim. Sci..

[48] Xue B., Chai S.T., Liu S.J., Wang W.B. (1994). Study on the protein requirement of growing yaks. Chinese Qinghai. J. Anim. Vet..

[49] Hu L.H., Xie A.Y., Han X.T. (1994). Study on the body surface areas of growing yaks and cattle. Chin. J. Anim. Sci..

[50] Markert C.L. (1984). Biochemistry and function of lactate dehydrogenase. Cell Biochem. Funct..

[51] Lin Y.Q., Wang G.S., Feng J., Huang J.Q., Xu Y.O., Jin S.Y., Li Y.P., Jiang Z.R., Zheng Y.C. (2011). Comparison of enzyme activi-ties and gene expression profiling between yak and bovine skeletal muscles. Livest. Sci..

[52] Gnecchi-Ruscone G.A., Abondio P., De Fanti S., Sarno S., Sherpa M.G., Sherpa P.T., Marinelli G., Natali L., Di Marcello M., Peluzzi D. (2018). Evidence of Polygenic Adaptation to High Altitude from Tibetan and Sherpa Genomes. Genome Biol. Evol..

[53] Amos W., Harwood J. (1998). Factors affecting levels of genetic diversity in natural populations. Philos. Trans. R. Soc. B Biol. Sci..

[54] Pritchard J., Di Rienzo A. (2010). Adaptation not by sweeps alone. Nat. Rev. Genet..

[55] Höllinger I., Pennings P.S., Hermisson J. (2019). Polygenic adaptation: From sweeps to subtle frequency shifts. PLoS Genet..

[56] Ding X., Yang C., Bao P., Wu X., Pei J., Yan P., Guo X. (2019). Population genetic variations of the matrix metalloproteinases-3 gene revealed hypoxia adaptation in domesticated yaks (*Bos grunniens*). Asian-Australas. J. Anim. Sci..

[57] Dolt K.S., Mishra M.K., Karar J., Baig M.A., Ahmed Z., Pasha M.Q. (2007). cDNA cloning, gene organization and variant specific expression of HIF-1α in high altitude yak (*Bos grunniens*). Gene.

[58] Allen M.S., Bradford B.J., Oba M. (2009). Board-Invited Review: The hepatic oxidation theory of the control of feed intake and its application to ruminants. J. Anim. Sci..

[59] Weimer P.J., Russell J.B., Muck R.E. (2009). Lessons from the cow: What the ruminant animal can teach us about consolidated bio-processing of cellulosic biomass. Bioresour. Technol..

[60] Moon Y.A., Horton J.D. (2003). Identification of two mammalian reductases involved in the two-carbon fatty acyl elongation cascade. J. Biol. Chem..

[61] Li Y., Trojer P., Xu C.F., Cheung P., Kuo A., Drury III W.J., Qiao Q., Neubert T.A., Xu R.M., Gozani O. (2009). The target of the NSD family of histone lysine methyltransferases depends on the nature of the substrate. J. Biol. Chem..

[62] Ding Y., Liu J., Xu Y., Dong X., Shao B. (2020). Evolutionary Adaptation of Aquaporin-4 in Yak (*Bos grunniens*) Brain to High-Altitude Hypoxia of Qinghai-Tibetan Plateau. High Alt. Med. Biol..

[63] Wang J., Shi Y., Elzo M.A., Dang S., Jia X., Lai S. (2017). Genetic diversity of ATP8 and ATP6 genes is associated with high-altitude adaptation in yak. Mitochondrial DNA Part A.

[64] Wang H., Chai Z., Hu D., Ji Q., Xin J., Zhang C., Zhong J. (2019). A global analysis of CNVs in diverse yak populations using whole-genome resequencing. BMC Genom..

[65] Shi Y., Hu Y., Wang J., Elzo M.A., Yang X., Lai S. (2017). Genetic diversities of MT-ND1 and MT-ND2 genes are associated with high-altitude adaptation in yak. Mitochondrial DNA Part A.

[66] Guang-Xin E., Yang B.-G., Zhu Y.-B., Duang X.-H., Basang W.-D., Luo X.-L., An T.-W. (2020). Genome-wide selective sweep analysis of the high-altitude adaptability of yaks by using the copy number variant. 3 Biotech.

[67] Somero G.N. (2005). Linking biogeography to physiology: Evolutionary and acclamatory adjustments of thermal limits. Front Zool..

[68] Gracey A.Y., Chaney M.L., Boomhower J.P., Tyburczy W.R., Connor K., Somero G.N. (2008). Rhythms of Gene Expression in a Fluctuating Intertidal Environment. Curr. Biol..

[69] Qi X., Zhang Q., He Y., Yang L., Zhang X., Shi P., Yang L., Liu Z., Zhang F., Liu F. (2018). The Transcriptomic Landscape of Yaks Reveals Molecular Pathways for High Altitude Adaptation. Genome Biol. Evol..

[70] Lisy K., Peet D.J. (2008). Turn me on: Regulating HIF transcriptional activity. Cell Death Differ..

[71] Webb J.D., Coleman M., Pugh C.W. (2009). Hypoxia, hypoxia-inducible factors (HIF), HIF hydroxylases and oxygen sensing. Cell. Mol. Life Sci..

[72] Xiong X., Fu M., Lan D., Li J., Zi X., Zhong J. (2015). Yak response to high-altitude hypoxic stress by altering mRNA expression and DNA methylation of hypoxia-inducible factors. Anim. Biotech..

[73] Semenza G.L., Wang G.L. (1992). A Nuclear factor induced by hypoxia via de novo protein synthesis binds to the human erythropoietin gene enhancer at a site required for transcriptional activation. Mol. Cell Biol..

[74] Schofield C., Ratcliffe P. (2004). Oxygen sensing by HIF hydroxylases. Nat. Rev. Mol. Cell Biol..

[75] Maxwell P.H. (2005). Hypoxia-inducible factor as a physiological regulator. Exp. Physiol..

[76] Zhao T.B., Ning H.X., Zhu S.S., Sun P., Xu S.X., Chang Z.J., Zhao X.Q. (2004). Cloning of hypoxia-inducible factor 1alpha cDNA from a high hypoxia tolerant mammal-plateau pika (*Ochotona curzoniae*). Biochem. Biophys. Res. Commun..

[77] Wang K., Yang Y., Wang L., Ma T., Shang H., Ding L., Han J., Qiu Q. (2015). Different gene expressions between cattle and yak provide insights into high-altitude adaptation. Anim. Genet..

[78] Xin J.-W., Chai Z.-X., Zhang C.-F., Zhang Q., Zhu Y., Cao H.-W., Ji Q.-M., Zhong J.-C. (2019). Transcriptome profiles revealed the mechanisms underlying the adaptation of yak to high-altitude environments. Sci. Rep..

